# Comparison of the Rate Constants of ^•^OH, SO_4_^•−^, CO_3_^•−^, Cl_2_^•−^, Cl^•^, ClO^•^ and H^•^ Reactions with Organic Water Contaminants

**DOI:** 10.3390/molecules30183741

**Published:** 2025-09-15

**Authors:** László Wojnárovits, Erzsébet Takács

**Affiliations:** Department of Surface Chemistry and Catalysis, Institute for Energy Security and Environmental Safety, HUN-REN Centre for Energy Research, Konkoly-Thege M. út 29–33, 1121 Budapest, Hungary; wojnarovits.laszlo@ek-cer.hu

**Keywords:** diffusion controlled rate constant, chemical reactivity controlled rate constant, correlations between rate constants, reduction potential

## Abstract

The reactions of 7 radicals, which play a key role in the degradation of water contaminants in Advanced Oxidation Processes, were discussed and compared. For evaluation of the reactivities and comparisons, the chemical reactivity rate constants were used, i.e., the rate constant that would be measured if diffusion of the species was not rate-influencing (*k*_chem_). By selecting appropriate diffusion-controlled rate constants (*k*_diff_) and using the measured rate constant (*k*) values, *k*_chem_ was calculated by the Noyes equation: 1/*k* = 1/*k*_chem_ + 1/*k*_diff_. When *k* and *k*_diff_ were close to each other, *k*_chem_ was much higher than *k*. log *k*_chem_ values showed good correlation in the cases of the ^•^OH-H^•^, SO_4_^•−^-^•^OH, and Cl_2_^•−^-CO_3_^•−^ radical pairs, moderate correlation was found in the case of the CO_3_^•−^-ClO^•^ pair. The correlations may reflect, at least partly, similar reaction mechanisms, and allow estimation of unknown rate constant values. No correlation was found for the ^•^OH-Cl^•^ pair; this indicates highly different reaction mechanisms. The reactivity of oxidizing radicals decreases with decreasing reduction potential in the order: Cl^•^ > ^•^OH > SO_4_^•−^ > Cl_2_^•−^ > CO_3_^•−^ > ClO^•^. The reductive H^•^ reactions with organic molecules are similar to those of ^•^OH, although the H^•^ rate constants are 0.5–1 order of magnitude smaller.

## 1. Introduction

In Advanced Oxidation Processes (AOP) reactive inorganic radicals induce the degradation of harmful organic contaminants in water: in most cases, the hydroxyl radical (^•^OH) or the sulfate radical anion (SO_4_^•−^) is the primary initiating species [1]. Both radicals have strong oxidizing ability in reactions with practically all organic molecules. At the same time, by reacting with suitable partners, ^•^OH and SO_4_^•−^ may transform to less reactive radicals [2,3], e.g., in ^•^OH reaction with hypochlorite/hypochlorite ion (Reaction (1)) or carbonate ion/bicarbonate ion (2) chloride monoxide radical (ClO^•^) and carbonate radical anion (CO_3_^•−^), respectively, form [4,5]:

(1) OH•+ClO−/HClO→OH−/H2O+ClO• 9.0 × 109/3.0 × 109 mol−1 dm3 s−1

(2) OH•+CO32−/HCO3−→OH−/H2O+CO3•− 3.9 × 108/8.5 × 106 mol−1 dm3 s−1

In the reaction between chloride ion (Cl^−^) and ^•^OH, dichloride radical anion (Cl_2_^•−^) is produced in a multistep process (see later), in the process chlorine atoms (Cl^•^) are also intermediates [6]. SO_4_^•−^ in reactions with CO_3_^2−^/HCO_3_^−^ and Cl^−^ produces carbonate radical ion (CO_3_^•−^) or chlorine radicals, respectively. In some AOP, such as sonolysis or VUV, H-atoms are also formed as reactive intermediates together with ^•^OH.

In this study, we compare the rate constants of 7 radicals, ^•^OH, SO_4_^•−^, CO_3_^•−^, Cl_2_^•−^, Cl^•^, ClO^•^, and H^•^ measured in reactions with organic pollutants in water. The reduction potential of all radicals *vs*. NHE, except one, H^•^, is positive; they are considered to be oxidizing radicals (Table 1). H^•^ reacting with higher oxidized metal ions can reduce them [7,8]. However, in reaction with organic molecules, H^•^ may behave similarly to the electrophile ^•^OH [8,9,10]. We discuss this question in more detail later on including also the effect of diffusion on the rate constant values [11,12].

The chemical reactivity and reaction mechanism of the 7 radicals show both similarities and important differences. In liquids, the chemical reactions take place after the reactants have approached each other by diffusion. Therefore, besides the chemical reactivity, diffusion can also strongly influence the reaction rate [11,12]. A novelty of the present work is the comparison of chemical reactivities that are not influenced by the different diffusion capabilities of the radicals.

## 2. The Diffusion Controlled Rate Constant

The ‘theoretical’ maximum value, the fully diffusion-controlled rate constant (*k*_diff_), is often estimated by the Smoluchowski equation [14]:

(3) $$k_{diff}=4\pi \left(D_{R•}+D_{org}\right)\left(r_{R•}+r_{org}\right)N\times 10^{3}\;\left({\mathrm{mol}}^{-1}\;{\mathrm{dm}}^{3}\;s^{-1}\right)$$

For using this equation, measured, or estimated values for the *D*_R•_ and *D*_org_ diffusion coefficients of the reacting radical and the organic molecule are needed. *r*_R•_ and *r*_org_ are the reaction radii of the corresponding species. *N* is Avogadro’s number. The estimated *k*_diff_ values for the radicals in aqueous solutions are collected in Table 2.

Ashton et al. [11] suggested using the reaction radius and diffusion coefficient of ^•^OH *r*_•OH_ = 0.22 × 10^−9^ m and *D*_•OH_ = 2.31 × 10^−9^ m^2^ s^−1^, respectively. For small aliphatic alcohol and aromatic molecules, Elliot et al. [12] and Ashton et al. [11] suggested *D*_org_ ≈ 0.5×10^−9^ m^2^ s^−1^ and *r*_org_ ≈ 0.30 × 10^−9^ m. With these values *k*_diff,•OH_ = 1.1 × 10^10^ mol^−1^ dm^3^ s^−1^. We assume that the diffusion-controlled rate constants do not depend greatly on the properties of organic molecules. The diffusion coefficient of ^•^OH (*D*_•OH_) is much higher than that of organic molecules (*D*_org_); therefore, mainly *D*_•OH_ determines the calculated value. In the case of organic molecules, there is some compensation. When the molecule is larger, the diffusion coefficient (*D*_org_) is smaller, and the reaction radius (*r*_org_) is larger. The near constancy of *k*_diff,•OH_ is supported by the results obtained analyzing the rate constants of ^•^OH reactions with a number of simple aromatic molecules [15]. The present analysis of rate constants, as we show later, also supports a near constancy.

The diffusion of the bulky SO_4_^•−^ should be slower than that of ^•^OH. Rickman and Mezyk [16] using Equation (3) calculated a *k*_diff,SO4•−_ of 7.36 × 10^9^ mol^−1^ dm^3^ s^−1^. Based on our analysis of rate constants, involving a large number of molecules in the investigation, we suggest *k*_diff,SO4•−_ ≈ 8 × 10^9^ mol^−1^ dm^3^ s^−1^ [17]. For the *k*_diff,H•_ and *k*_diff,Cl2•−_ we calculated 2.9 × 10^10^ mol^−1^ dm^3^ s^−1^ and 7.3 × 10^9^ mol^−1^ dm^3^ s^−1^, respectively, [18,19] using the radical radii and diffusion constants of Kazmierczak et al. [20]. For *k*_diff,CO3•−_we assumed the same value as for *k*_diff,Cl2•−_ [18]. Based on the diffusion-controlled rate constants for similar species and also on the range of rate constants for Cl^•^ and ClO^•^ reactions, *k*_diff,Cl•_ ≈ 2 × 10^10^ mol^−1^ dm^3^ s^−1^ and *k*_diff,ClO•_ ≈ 1 × 10^10^ mol^−1^ dm^3^ s^−1^, respectively, are suggested [18,21].

When two species, the reacting radical and the target molecule, approach each other by diffusion, the rate of the chemical step is determined by the physicochemical characteristics of the radical and the target molecule. The rate constant of chemical reactivity controlled reaction [12], i.e., the rate constant that would be measured if diffusion of the species was not rate-influencing, *k*_chem_, can be estimated according to the Noyes equation [22]:

(4) $$\frac{1}{k}=\frac{1}{k_{diff}}+\frac{1}{k_{chem}}$$

**Figure 1:**
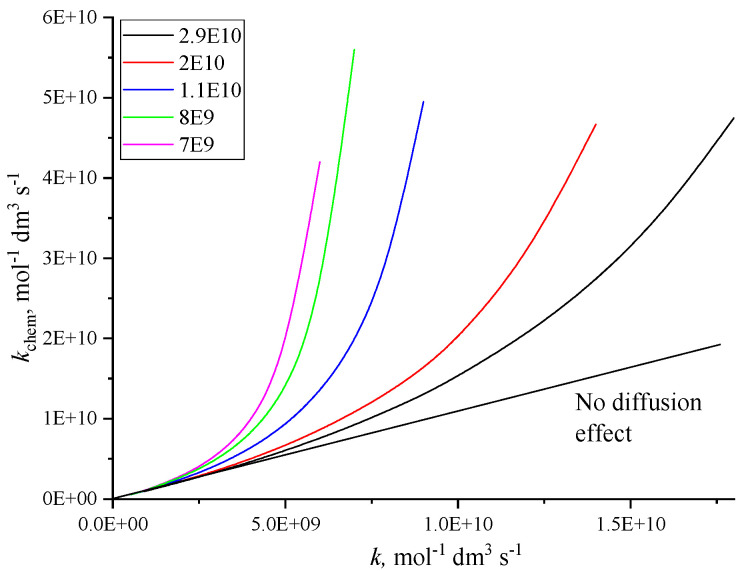
Effect of diffusion on the experimentally determined rate constants (*k*). The *k*_chem_ values were calculated using the Noyes equation (Equation (4)) and selecting several *k*_diff_ values shown in the legend.

*k* and *k*_diff_ are the measured and diffusion controlled rate constant. In Figure 1 we show the connection between observed and chemical reactivity controlled rate constants calculated for several *k*_diff_ values. When *k* is close to *k*_diff_, Equation (4) reveals high reactivity (high *k*_chem_): with *k*_diff_ = 1 × 10^10^ mol^−1^ dm^3^ s^−1^ and *k* = 5 × 10^9^ mol^−1^ dm^3^ s^−1^, *k*_chem_ is calculated to be 1 × 10^10^ mol^−1^ dm^3^ s^−1^. For rate constant evaluation, actually for the correction of the observed rate constant in order to obtain the rate constant of the chemical reaction, this equation is recommended.

Inorganic radicals may react in three different ways with organic molecules: single electron transfer (SET), radical adduct formation (RAF), and H-atom abstraction from saturated parts of molecules (HAT). The ratios of the three processes depend on the reaction partners and on the reaction conditions. Sometimes all three mechanisms are operative.

## 3. Database

In the last 10 years, we published review papers on the rate constants of SO_4_^•−^, CO_3_^•−^, Cl_2_^•−^, Cl^•^, and ClO^•^ reactions with organic molecules. We also reviewed the reactions of ^•^OH with pesticides and antibiotics. In the present evaluation, we refer to the rate constant data collected in these papers (Table 3 and Table 4) and not to the original works, because of the large number, more than 500, of original works used for creating the review papers. Moreover, these publications contain many averaged or recommended values. Besides these works, we use data from other compilations as well. *k*_H•_ data were mostly taken from the NDNR/NIST database, and from the works of Madden and Mezyk [8] and Homlok et al. [10]. We tried to select “reliable” rate constants. It is an important question: which rate constant is reliable? When several experimental data were available, which did not differ considerably, we used the average. We disregarded values, which highly exceeded the diffusion-controlled rate constant, and were rather careful about the calculated ones. The calculated values, when there was a possibility for comparison, often differed by more than one order of magnitude from the experimental ones.

### 3.1. Connection Between Hydroxyl Radical and Hydrogen Atom Rate Constants

^•^OH can be produced in a large number of ways including hydrogen peroxide photolysis, photocatalysis, sonochemistry, VUV irradiation or radiolysis of aqueous solutions [1]. This radical may also have an important role in the SO_4_^•−^ based techniques, where SO_4_^•−^ reacts with H_2_O/OH^−^ to supply ^•^OH (Reactions (5) and (6)) [43]:

(5) SO4•−+H2O→SO42−+OH•+H+ k = 6.6 × 102 mol−1 dm3 s−1

(6) SO4•−+OH−→SO42−+OH• k = 7 × 107 mol−1 dm3 s−1

In experiments with sulfate radical anions, below pH 9 SO_4_^•−^, above pH 11 ^•^OH dominates the reaction system, and between the two pHs, the two radicals coexist [43].

H^•^ forms with a yield equivalent to ^•^OH in water splitting during VUV photolysis or sonolysis. In water radiolysis, the H^•^ yield is smaller (0.06 μmol J^−1^ absorbed energy) than the yields of the other two primary radical species, hydroxyl radical and hydrated electron (0.28 μmol J^−1^ for each). However, at low pH (<3–4), the hydrated electron (e_aq_^−^) in reaction with H_3_O^+^ (Reaction (7)) transforms to H^•^, and the yield goes up to 0.34 μmol J^−1^ [44].

(7) $$e_{aq}^{-}+H_{3}O^{+}\to H^{•}+H_{2}O$$

**Figure 2:**
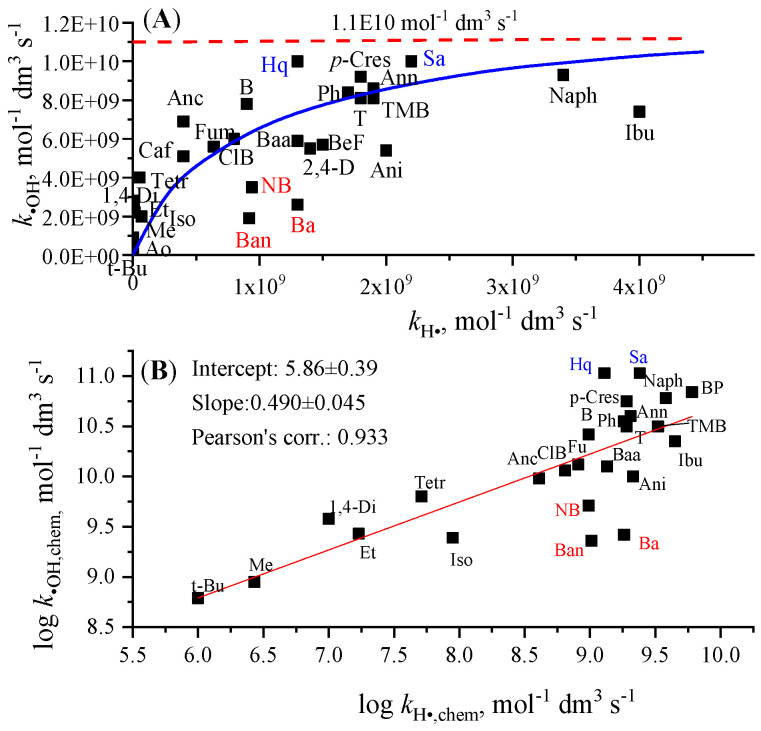
Relation between *k*_•OH_ and *k*_H•_ (**A**), and log *k*_•OH,chem_ and log *k*_H•,chem_ (**B**) (see abbreviations in Table 3 and Table 4).

We show the *k*_•OH_ values in Figure 2A as a function of *k*_H•_ data. Based on the figure, there is certainly a correlation between the two rate constants: as *k*_•OH_ increases, *k*_H•_ also tends to increase. The increase is strong at low H-atom rate constants. At high *k*_H•_’s, *k*_•OH_ shows a tendency to saturate at a constant value of 1.1 × 10^10^ mol^−1^ dm^3^ s^−1^. This value is the diffusion-controlled rate constant of ^•^OH (Table 2). The effect of diffusion on *k*_H•_ is much smaller, since *k*_H•_’s are lower than *k*_•OH_’s, and also *k*_diff,H•_ is higher, 2.9 × 10^10^ mol^−1^ dm^3^ s^−1^, than *k*_diff,•OH_ 1.1 × 10^10^ mol^−1^ dm^3^ s^−1^ (Table 2). The measured uncorrected *k*_•OH_ values are, on average, 5.2 times higher than the *k*_H•_ values (Table 3 and Table 4). This difference is much higher, 14.7, if we consider the values corrected for the effect of diffusion. Figure 2B shows the logarithms of the corrected rate constants collected in Figure 2A. The figure reflects a linear correlation between log *k*_•OH_,_chem_ and *k*_H•,chem_ with a slope of 0.490 ± 0.045.

Both ^•^OH and H^•^ react with organic molecules, predominantly in radical adduct formation (RAF) at the double bonds or in H-atom abstraction from the saturated parts of molecules (HAT). There is no reliable evidence in the literature for electron transfer (SET) reactions. In most publications ^•^OH is considered to be a non-selective radical in reaction with organic molecules e.g., [41,45,46,47]. As we will show, this is certainly not true. The selectivity is obvious when we compare the *k*_•OH_’s of aromatic molecules having different substituents [15]. When electron donating groups are on the ring (-OH, -CH_3_, NH_2_) the rate constants of ^•^OH reactions are higher (*k*_•OH_ ≈ 8 × 10^9^ mol^−1^ dm^3^ s^−1^) than with electron withdrawing substituents (-Cl, -NO_2_, -COOH, ~3.5 × 10^9^ mol^−1^ dm^3^ s^−1^) (Table 3). This difference is much higher in the case of chemical reactivity rate constants, *k*_•OH_,_chem_ ≈ 2.9 × 10^10^ mol^−1^ dm^3^ s^−1^ and ~5.1 × 10^9^ mol^−1^ dm^3^ s^−1^, respectively. The closeness of the diffusion-controlled rate constant to the measured values masks the large selectivity. The selectivity is also observed when the ^•^OH reacts with molecules having electron-rich and electron-poor parts (we call it inner selectivity). Due to the electrophile character, ^•^OH preferably reacts with the electron-rich parts. When ^•^OH approaches an aromatic ring, it senses the charge distribution on the ring and adds to the higher electron density places. For instance, in phenol, the OH group increases the electron density on the ring in *ortho*- and *para* positions: ^•^OH preferably adds to these positions (2 × 25% and 34%), while *meta*- and *ipso* additions have a smaller frequency (2 × 4% and 8%) [48,49,50,51].

The correlation between the *k*_•OH_ and *k*_H•_ reflects that H^•^, like ^•^OH, also has electrophile character in reactions with organic molecules, as it has been suggested previously [9,10,52,53]. In the fitting (Figure 2B), the values measured for benzaldehyde, benzoic acid (neutral), and nitrobenzene were disregarded. In these cases, our considerations [10] and also end product results suggest participation of additional reaction channels in H^•^ reaction, in addition to the ring or H-abstraction; most probably H^•^ directly reacts with the substituent on the ring [54,55], thus increasing the reactivity. In the nitrobenzene reaction, this additional channel may be direct NO_2_ elimination [10]. We also disregarded the values belonging to hydroquinone and salicylic acid. Their published *k*_•OH_ (1 × 10^10^ mol^−1^ dm^3^ s^−1^) is close to *k*_•OH,diff_ (1.1 × 10^10^ mol^−1^ dm^3^ s^−1^) making *k*_•OH,chem_ calculation unreliable.

Although much less data is available on the rate constants and mechanisms of H^•^ reactions, H^•^, similarly to ^•^OH, also preferably reacts at the electron-rich places of molecules (inner selectivity). In addition to toluene and phenol, a similar *ortho-para* directing effect was suggested with both radicals [10,56]. As regards H-abstraction (HAT), both radicals predominantly react with the weakest H-bonds in the molecules, e.g., abstraction of tertiary H of isopropanol is about 6 times more frequent than that of primary hydrogens (bond strengths, 384 and 389 kJ mol^−1^, respectively, [57]).

### 3.2. Rate Constants of Sulfate Radical Anion Reactions with Organic Molecules

Literature suggests a brilliant future for SO_4_^•−^ based purification technologies [28,43,45,58]. In industry, these radicals are produced by activation of peroxidisulfate (S_2_O_8_^2−^) or peroxymonosulfate (HSO_5_^−^), e.g., by heat, transition metal ions (Fe^2+^, Co^2+^), or UV photons [59,60]. When the activation takes place using ionizing radiation, e.g., for rate constant determination, e_aq_^−^ induces the formation of SO_4_^•−^ (Reaction (8)):

(8) $$S_{2}O_{8}^{2-}+e_{aq}^{-}\to {SO}_{4}^{2-}+{SO}_{4}^{•-}$$

Due to their industrial applicability, SO_4_^•−^ reactions have been frequently investigated in laboratory and semi-industrial experiments: many rate constants are available for SO_4_^•−^ reactions [17,61]. The majority of them were determined in pulsed radiolysis or laser flash photolysis experiments. The highest values are in the 6 × 10^9^–8 × 10^9^ mol^−1^ dm^3^ s^−1^ range when determined by transient techniques, and the values measured in different laboratories are close to each other. When *k*_SO4•−_’s are measured using competitive techniques, the values show large scatter. The greater uncertainty may be due to the complex reaction system. In stationary experiments, the radical lifetime is much longer than in pulse radiolysis or laser flash photolysis. Therefore, a longer time is available for SO_4_^•−^ to transform to ^•^OH in reaction with H_2_O/OH^−^ (Reactions (5) and (6)). In laboratory experiments, radical scavengers, e.g., *tert*-butanol, are used for the separation of SO_4_^•−^ and ^•^OH reactions. SO_4_^•−^ reacts with a three-orders-of-magnitude-smaller rate constant with *tert*-butanol than ^•^OH [2], 7.4 × 10^5^ and 6.2 × 10^8^ mol^−1^ dm^3^ s^−1^, respectively (Table 3).

**Figure 3:**
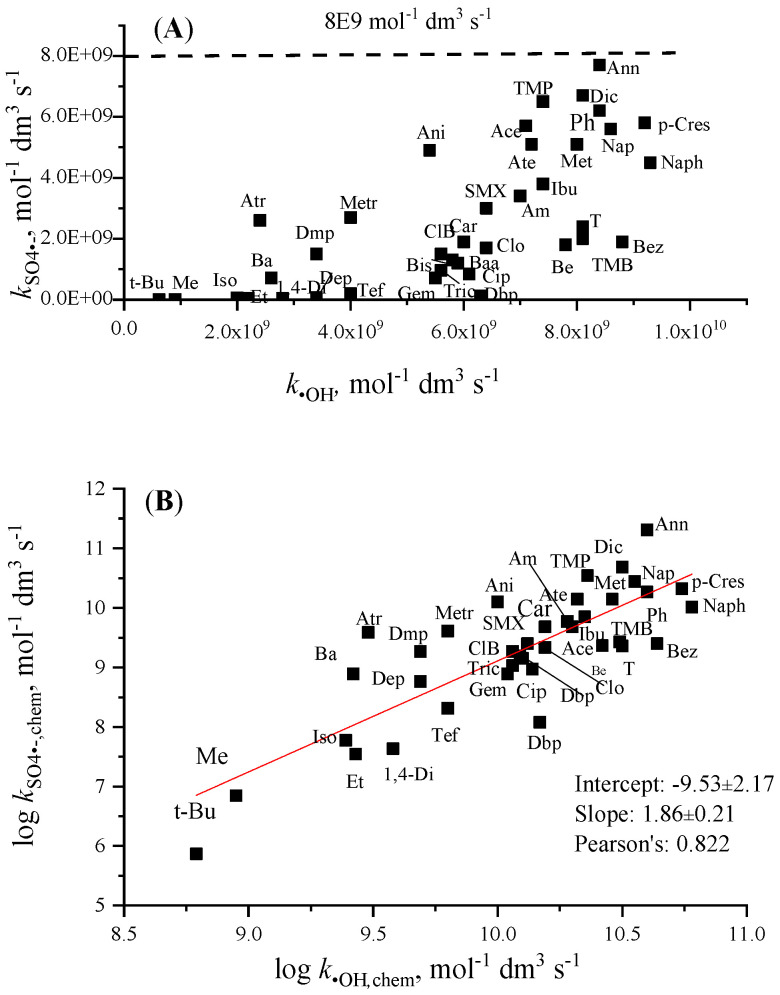
Relation between *k*_•OH_ and *k*_SO4•−_ (**A**), and log *k*_•OH,chem_ and log *k*_SO4•−,chem_ (**B**) (see abbreviations in Table 3 and Table 4).

In Table 3 and Table 4 the highest *k*_SO4•−_ values are 30–50% below the highest *k*_•OH_ data. This difference is partly due to the higher diffusion-controlled rate constant for ^•^OH reactions than for SO_4_^•−^ reactions, 1.1 × 10^10^ and 8 × 10^9^ mol^−1^ dm^3^ s^−1^, respectively (Table 2). SO_4_^•−^, similarly to ^•^OH, is a highly electrophile reactant; however, SO_4_^•−^ is more selective than ^•^OH [17,47]. This higher selectivity is reflected by the wider range of rate constants for SO_4_^•−^ than for ^•^OH (Figure 3A). The slope (1.86) of the log *k*_SO4•-,chem_ − *k*_•OH,chem_ plot (Figure 3B), much higher than 1, also shows greater molecule structure dependence of SO_4_^•−^ reactions. For aromatics, the highest values are for molecules with electron-releasing substituents (e.g., a methoxy group) on the ring; these values are close to the diffusion-controlled limit. Therefore, as Steenken et al. [62] noted, the rate constants of methoxylated benzenes show hardly any structural dependence. On the contrary, nitrobenzene with the electron-withdrawing NO_2_ substituent practically does not react with SO_4_^•−^. Low values were measured for fluorobenzene, chlorobenzene, benzoic acid, and benzaldehyde; in these molecules, the electron-withdrawing substituent decreases reactivity [17]. SO_4_^•−^ similarly to ^•^OH reacts with saturated molecules by H-abstraction reaction (HAT), the *k* values here also show high bond-strengths effect. The rate constants of abstraction reactions from alcohols in Table 3 are, on average, two orders of magnitude smaller for SO_4_^•−^ than for ^•^OH.

In contrast to ^•^OH, in most papers the basic reaction between aromatic molecules and SO_4_^•−^ is suggested to be SET [17]. The positively charged aromatic ring, formed during the charge transfer, in reaction with a water molecule and by H^+^ elimination, transforms to a hydroxycyclohexydienyl intermediate, similar to ^•^OH addition to the aromatic ring. However, some papers also propose the RAF mechanism. In this mechanism, SO_4_^2−^ elimination from the adduct is followed by H_2_O/H^+^ addition/elimination, yielding also hydroxycyclohexydienyl radical. The question is the lifetime of SO_4_^•−^ adduct. Some papers suggest a very short lifetime and assume that transient techniques cannot detect it [62,63]. The authors in other papers claim that they observed the adduct and report on the characteristics of this short-lived intermediate [64,65,66,67]. The correlation between the rate constants of ^•^OH and SO_4_^•−^ reactions (Figure 3) may suggest, at least partly, a similar mechanism for the two reactions.

### 3.3. Correlation Between Carbonate Radical Anion and Dichloride Radical Anion Rate Constants

Wastewaters generally contain high concentrations of carbonate/bicarbonate (CO_3_^2−^/HCO_3_^−^, p*K*_a_ 10.32) and chloride ions (Cl^−^). ^•^OH readily reacts with CO_3_^2−^/HCO_3_^−^ forming CO_3_^•−^ (Reaction (2)) [4,47,68]. This reaction is used for CO_3_^•−^ production under laboratory conditions. In the presence of carbonate/bicarbonate in sulfate-based technologies and laboratory experiments with SO_4_^•−^ (and several other radicals, e.g., Cl_2_^•−^, Cl^•^), CO_3_^•−^ is also produced. This generation technique was used in rate constant determination, e.g., in laser flash photolysis experiments of Umschlag and Herrmann [69] and Dell’Arciprete et al. [70]. Similarly to CO_3_^•−^, Cl_2_^•−^ can also be produced in radical transfer involving ^•^OH or SO_4_^•−^. However, the mechanism is complex [71]. In Scheme (9), we show a simplified mechanism with ^•^OH as the initiating radical. Cl_2_^•−^ forms after several equilibrium processes, finally in the Cl^•^ reaction with Cl^−^.

(9) +H+                    -H2O  +Cl-OH+Cl−⇄•ClOH•−⇄(HOClH)•⇄Cl•⇄Cl2•−                               +H+                    -H2O  +Cl-

Due to participation of H^+^ in one of the equilibria, the Cl_2_^•−^ abundance is strongly pH dependent: below pH 5, the dominant species is Cl_2_^•−^, above this pH ^•^OH dominates [43]. SO_4_^•−^ reaction with Cl^−^ directly produces Cl^•^ [72].

Approximately 300 rate constants are reported in the literature for carbonate radical anion reactions with organic molecules. We estimate the same number of rate constants for the reactions of the dichloride radical anion. There are papers that publish and compare CO_3_^•−^ rate constants for a large number of contaminants, e.g., Umschlag and Herrmann [69], Canonica et al. [73], Dell’Arciprete et al. [70], Jasper and Sedlak [74]; Wols et al. [75]. Many rate constants are available on Cl_2_^•−^ reactions, as well [76,77,78,79]. Lei et al. [21] published Cl_2_^•−^ and Cl^•^ reaction rate constants for 88 organic contaminants. Critical evaluations of the available rate constants for CO_3_^•−^ and Cl_2_^•−^ reactions are published in review papers of Wojnárovits et al. [4] and Wojnárovits and Takács [19].

CO_3_^•−^ and Cl_2_^•−^ are highly selective radicals [4,19,47]. They react with aliphatic molecules without double bonds with small *k*_CO3•−_ and *k*_Cl2•−_ values in the 10^2^–10^5^ mol^−1^ dm^3^ s^−1^ range. This range is 4–6 orders of magnitude smaller than the range for ^•^OH, 10^8^–10^9^ mol^−1^ dm^3^ s^−1^, or SO_4_^•−^, 10^5^–10^7^ mol^−1^ dm^3^ s^−1^, reactions. However, the rate constants of reactions with amine and sulfur compounds are higher (10^5^–10^6^ mol^−1^ dm^3^ s^−1^, [80]) than those with simple alcohols. The *k*_CO3•−_ of cysteine reaction is 1.9 × 10^8^ mol^−1^ dm^3^ s^−1^ (Table 3). For diethylamine and piperidine Elango et al. [80] reported 3.8 × 10^6^ and 3.3 × 10^6^ mol^−1^ dm^3^ s^−1^, respectively. The rate constants of Cl_2_^•−^ reactions are especially high, they are in the 10^7^–10^9^ mol^−1^ dm^3^ s^−1^ range, in reaction with organic sulfides and sulfoxides (e.g., [81,82,83]). *k*_Cl2•−_ in cysteine reaction is 8.5 × 10^8^ mol^−1^ dm^3^ s^−1^ (Table 3). Sulfur radicals have a high ability to stabilize in three-bonded complexes, e.g., S∴Cl, with two electrons on σ* bonding and one on σ* antibonding orbital. These complexes easily dimerize. We show these Reactions ((10) and (11)) on the example of a thioether (R^1^R^2^S)

(10) $${Cl}_{2}^{•-}+R^{1}R^{2}S↔R^{1}R^{2}S∴Cl+{Cl}^{-}$$

(11) $$R^{1}R^{2}S∴Cl+R^{1}R^{2}S↔R^{1}R^{2}S∴SR^{1}R^{2}+{Cl}^{-}$$

Both CO_3_^•−^ and Cl_2_^•−^ have smaller reactivity with aromatic molecules as ^•^OH or SO_4_^•−^ [4,19,21,47]. In CO_3_^•−^ reactions, the highest values are in the 1 × 10^6^–1 × 10^9^ mol^−1^ dm^3^ s^−1^ range (Table 3 and Table 4). This range for Cl_2_^•−^ is 1 × 10^7^–2 × 10^9^ mol^−1^ dm^3^ s^−1^. Even the highest values are much smaller than the diffusion-limited value (7.3 × 10^9^ mol^−1^ dm^3^ s^−1^ for both radical anions, Table 2), diffusion has little influence on the measured values. Among the aromatic molecules, benzene and benzene derivatives with electron withdrawing substituents have especially small reactivity with CO_3_^•−^ and Cl_2_^•−^, the rate constants are in the 10^4^–10^5^ mol^−1^ dm^3^ s^−1^ range. The electron donating –CH_3_ group in toluene just slightly increases the reactivity. Phenol and phenolate have relatively high rate constants of 5 × 10^6^ and 6 × 10^7^ mol^−1^ dm^3^ s^−1^, respectively, in CO_3_^•−^ reactions [4]. Electron donating substituents highly increase *k*_CO3•−_ in phenols and the values may increase to 10^9^ mol^−1^ dm^3^ s^−1^. Anilines represent special cases [84]. The *k*_CO3•−_ of aniline is high 4.1 × 10^8^ mol^−1^ dm^3^ s^−1^ (average of 7 determinations, [4]), *k*_Cl2•−_ is 6 × 10^7^ mol^−1^ dm^3^ s^−1^ (Table 3). Methyl and ethyl substituents on the –NH_2_ group increase the rate constants to the 10^9^ mol^−1^ dm^3^ s^−1^ range [73].

**Figure 4:**
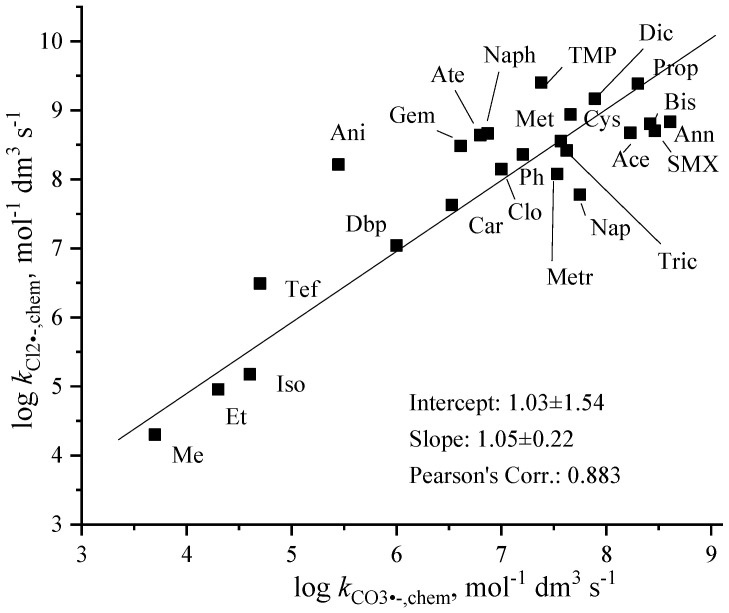
Relation between log *k*_CO3•−,chem_ and log *k*_Cl2•−,chem_ (see abbreviations in Table 3 and Table 4).

There is a relatively good linear dependence of the log *k*_Cl2•−,chem_ data on the *k*_CO3•−,chem_ values (Figure 4). The slope value, 1.05 ± 0.22, suggests similar reactivity of the two radicals with organic molecules. We mention that in the literature Cl_2_^•−^ is suggested to be somewhat more reactive as CO_3_^•−^ [19,47].

There is no agreement on the mechanism of CO_3_^•−^ reactions in the relevant publications [47,73,80,85]: all the three mechanisms, SET, RAF and HAT, may participate. Important to mention here, in the reaction of molecules with amino group, H-atom abstraction from −NH_2_ or −NHR- may have special importance. This mechanism is suggested for both, aliphatic amines like diethylamine, or for aniline and N-methylaniline [47,80]. As regards RAF, no adduct was detected in reaction with double bonded compounds, probably due to instability of adducts [47]. In former works SET mechanism was preferred in explanation of experimental results. However, thermodynamic calculations of Li et al. [47] suggest low contribution of SET. According to them RAF and HAT are the main reaction pathways of CO_3_^•−^ reactions.

In Cl_2_^•−^ reaction with aromatic molecules SET is suggested to be the basic interaction. Aliphatic olefins may react by RAF mechanism (Reaction (12)) giving chlorinated products [76,86]:

(12) $${Cl}_{2}^{•-}+R^{1}HC=CHR^{2}\to \left[R^{1}HC^{•}-\left({Cl}_{2}^{-}\right)HCR^{2}\right]\to R^{1}HC^{•}-ClHCR^{2}+{Cl}^{-}$$

The rate constants of CO_3_^•−^ and Cl_2_^•−^ reactions are generally orders of magnitude smaller than those of the ^•^OH reactions, resulting in longer lifetime for CO_3_^•−^ and Cl_2_^•−^, than for ^•^OH. In natural waters, the average CO_3_^•−^ concentration is c.a. 2–3 orders of magnitude higher than that of ^•^OH [73,74]. The high CO_3_^2−^/HCO_3_^−^ and Cl^−^ concentrations in natural waters compensate for the low *k* values [4]. These radicals play a very important role in the degradation of organic pollutants in different AOPs. Knowledge of reactivities and especially selectivity helps to understand and model the reactions taking place in natural waters or in the air droplets.

### 3.4. Chlorine Atom Reactions

In the ^•^OH + Cl^−^ reaction (Scheme (9)), on the way to Cl_2_^•−^ production Cl^•^ is an intermediate. When ^•^OH is used for the investigation of Cl^•^ reactions, both Cl^•^ and Cl_2_^•−^ react with the target molecules [71,87,88]. Reactions of Cl^•^ and Cl_2_^•−^ with water molecules ((13), (14)) also complicate the system:

(13) $${Cl}^{•}+H_{2}O\to products\;k\left[H_{2}O\right]\;=\;2.5\;\times \;10^{5}\;s^{-1}$$

(14) $${Cl}_{2}^{•-}+H_{2}O\to HOClH+{Cl}^{-}\;k\left[H_{2}O\right]\;=\;1300\;s^{-1}$$

The complex reaction system makes the rate constant measurements tiresome and decreases accuracy. Another technique for Cl^•^ production in laboratory experiments is photolysis of chloroacetone (Reaction (15)) [21,64,89].

(15) $$CH_{3}COCH_{2}Cl+h\nu \to {\left[CH_{3}COCH_{2}Cl\right]}^{*}\to CH_{3}COCH_{2}^{•}+{Cl}^{•}$$

Cl^•^ reactions with organic molecules show similarity to ^•^OH reactions, albeit Cl^•^ seems to be less selective as ^•^OH [23]. Due to uncertainty problems, it is difficult to determine which types of molecules are preferred in Cl^•^ reactions. However, the rate constants of Cl^•^ reactions with aromatic molecules are high, they are in the 10^9^–10^10^ mol^−1^ dm^3^ s^−1^ range, and the values are much higher than the rate constants of ^•^OH. This is partly due to the higher diffusion-limited rate constant value for Cl^•^, than for ^•^OH: ~2 × 10^10^ and 1.1 × 10^10^ mol^−1^ dm^3^ s^−1^, respectively (Table 2).

**Figure 5:**
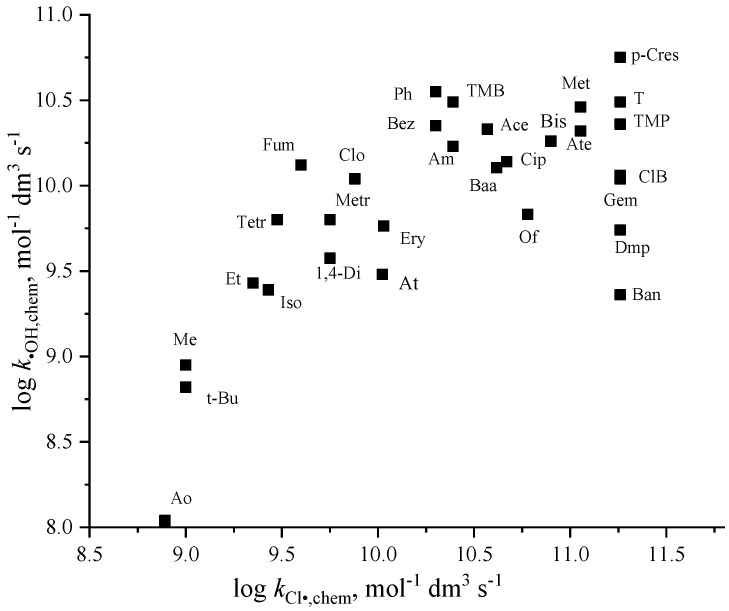
Relation between log *k*_•OH,chem_ and log *k*_Cl•,chem_ (see abbreviations in Table 3 and Table 4).

For saturated molecules, Buxton et al. [6] reported a definite correlation between *k*_Cl•_ and *k*_•OH_. Cl^•^ reacts with these molecules with rate constants in the 10^8^–10^9^ mol^−1^ dm^3^ s^−1^ range [6,90,91]. The reactions occur with the HAT mechanism. In reactions with alcohols, there is a preference for Cl^•^ reactions at the O-H group, rather than abstraction from C-H. Gilbert et al. [90] and Buxton et al. [6] assumed SET from the O-H group as a starting step of H-elimination. Correlation between Cl^•^ and ^•^OH rate constants is not obvious in reactions with double-bonded compounds (Figure 5). In the case of lower values, the RAF mechanism, for the higher ones, the SET mechanism has been suggested [21,92]. The latter mechanism was proved by theoretical calculations and by observing the radical cation intermediate in transient measurements [21,91]. Lei et al. [21], using the chloroacetone technique for Cl^•^production, published rather high values approaching or exceeding the diffusion-controlled limit for a few compounds. They explained these results in terms of SET, assuming that the two species do not necessarily diffuse and encounter in the solvent cage: transfer may occur when the reacting species are not in close contact. They also mention another possible explanation: diverse reaction sites are involved in the process, and the sum of them may exceed the diffusion-controlled value. These explanations are insufficient on theoretical grounds, and we cannot rule out the possibility of some systematic error. Buxton, who developed this technique, pointed out that the purity of chloroacetone significantly influenced the measured kinetics, a fact that seems to have been missed by many subsequent authors [6,91].

### 3.5. Chlorine Monoxide Radical Reactions

Chlorine monoxide (ClO^•^) plays an important role in the degradation of organic pollutants in waters containing Cl^−^ [5]. Cl^•^, Cl_2_^•−^, and ClO^•^ are the basic intermediates of hypochlorite (HOCl/OCl^−^) photolysis [23], which is considered to be an emerging AOP technology [93,94,95,96]. Due to the low reduction potential (*E*^0^(ClO^•^/ClO^−^) = 1.39 V), in the presence of HOCl/OCl^−^, ClO^•^ readily forms in radical transfer reactions with a number of radicals, including ^•^OH, Cl^•,^ or CO_3_^•−^ (Reaction (1)) [2,86]. HOCl/OCl^−^ is used for the chlorination of water; hypochlorite also forms when Cl_2_ is dissolved in water. In swimming pools, in the photoreaction of HOCl/OCl^−^, ^•^OH and Cl^•^ form (Reaction (16)), these radicals in further reaction may produce ClO^•^ [5].

(16) HOCl/OCl−+hν→Cl•+OH•/O− O−+H2O→OH•+OH−  pKa=11.9

Searching the literature, we found about 300 rate constants for ClO^•^ reactions with organic molecules: in most of cases, the source of ClO^•^ was HOCl/OCl^−^. In such systems, HOCl/OCl^−^ may also directly react with the solutes. However, this possibility is disregarded in most papers. Alfassi et al. [86] in their pioneering work used conditions (high pH) and model compounds when the disturbance exerted by HOCl/OCl^−^ was small. In pulse radiolysis experiments, they determined *k*_ClO•_ values for a few molecules, e.g., phenol-type compounds, dimethoxibenzenes. These rate constants, as the only ones determined applying the direct method, are used as reference values in the competitive techniques [97,98]. Since the rate constants for many simple molecules are very low, and it is difficult to measure low rate constants, the authors in several works tried to determine *k*_ClO•_’s by such theoretical methods as Density Functional Theory (DFT) and structure-reactivity (QSAR) calculations (e.g., [99,100]). These data should be used with caution since the values calculated for the same compound in different laboratories may differ by orders of magnitude.

ClO^•^ practically does not react with saturated molecules, e.g., alcohols, and many simple aromatic molecules, among them benzene [5]. Molecules that have activating (actually electron-rich) moieties have higher reactivities in ClO^•^ reactions [46,97], for instance, amine, or sulfite moieties, or in phenols/phenolates OH/O^−^. The rate constants of the latter compounds may be in the 10^8^–10^9^ mol^−1^ dm^3^ s^−1^ range. ClO^•^ reactions show definite electrophile character: electron-withdrawing substituents on the aromatic ring (e.g., in benzoates) decrease, electron-donating substituents (e.g., in anilines) increase the rate constants [94].

Based on the absence of reactions with aliphatic molecules, we assume that HAT is not involved in the reaction mechanism. Alfassi et al. [86], in the case of phenolates, suggested SET as the basic mechanism. However, in the most recent works, ClO^•^ addition to the unsaturated bonds is suggested as the main mechanism [99,100,101,102,103]. In RAF reactions of the substituted aromatic molecules, theoretical calculations suggest *ortho*/*para* preference (internal selectivity) [100].

**Figure 6:**
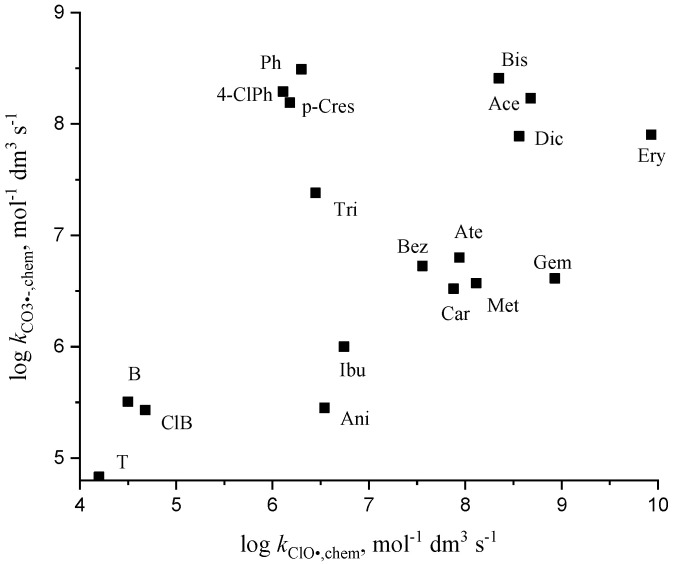
Relation between the log *k*_CO3•−,chem_ and log *k*_ClO•,chem_ (see abbreviations in Table 3 and Table 4).

In the case of ClO^•^ reactions, seeking correlations is extremely difficult because all smaller rate constants (e.g., for many aromatic molecules) are determined in quantum chemical calculations or in QSAR analysis. However, the *k*_ClO•_ values show some correlation with the rate constants of other low reactivity radicals, e.g., CO_3_^•−^ (Figure 6). It seems that both radicals have the same preferences in their reactions with organic molecules.

## 4. Comparison of the Reactivities of Different Radicals, Connection with the Reduction Potentials

The highest rate constants were determined for reactions of ^•^OH, SO_4_^•−,^ and Cl^•^ (Table 5). The reduction potentials of the ^•^OH/OH^−^, SO_4_^•−^/SO_4_^2−^, and Cl^•^/Cl^−^ redox couples are similar; they are between 2.4 V and 2.6 V (Table 1). However, the suggested diffusion-controlled rate constants are highly different; they are 1.1 × 10^10^, 8 × 10^9^ and ~2 × 10^10^ mol^−1^ dm^3^ s^−1^, respectively (Table 2). These differences are reflected in the measured values. The highest values for ^•^OH and SO_4_^•−^ approach these limits; in the case of Cl^•^, they may exceed the suggested diffusion-controlled rate constant; possible reasons are mentioned before. Except for the highest values, basically, the chemical reactivity determines the values of rate constants. This can be exemplified by the reactions of simple aromatics with ^•^OH, the rate constant reflects a definite electron releasing/electron withdrawing effect, although all the directly measured values are within half an order magnitude of the diffusion-controlled limit. A similar or even higher electron-releasing/electron-withdrawing effect is observed in the reactions of SO_4_^•−^. Such an effect is not obvious in the case of Cl^•^ reactions, partly due to the very high values.

Much fewer rate constants were measured for CO_3_^•−^, Cl_2_^•−^, and ClO^•^ than for the previous three radicals. The reduction potentials of these radicals are smaller, 1.78 V (pH 7), 2.1 V, and 1.39 V, respectively (Table 1). The diffusion-controlled rate constants are similar or smaller than for the radicals of the previous group: 7.3 × 10^9^, 7.3 × 10^9^, and ~1 × 10^10^ mol^−1^ dm^3^ s^−1,^ respectively (Table 2). The measured rate constants of these radicals, in just a few cases, approach the diffusion-limited values. Therefore, the rate constants are practically entirely controlled by the chemical reactivity. ClO^•^ does not react with aliphatic alcohols; in cases of CO_3_^•−^ and Cl_2_^•−^, some reactions were suggested, although with low rate constants.

H^•^ reactions show close similarity to the reactions of ^•^OH despite the very low negative reduction potential of H_aq_^+^/H^•^ = −1.9 V (Table 1) and the very high diffusion-controlled rate constant of 2.9 × 10^9^ mol^−1^ dm^3^ s^−1^ (Table 2). The rate constants of H^•^ reactions are about 0.5–2 orders of magnitude smaller than those of ^•^OH.

## 5. Conclusions

When the measured rate constant (*k*) is close to the diffusion-controlled constant (*k*_diff_), the chemically activated rate constant (*k*_chem_) is much higher than the experimental one. We suggest using *k*_chem_ instead of *k* when the reactivities of different radicals are compared, and also for comparison of the experimental results with the results of theoretical calculations.

H^•^ in reactions with organic molecules behaves similarly to ^•^OH. However, the measured *k*_•OH_ values on average are 5.2 times higher than the *k*_H•_ data for simple aromatic molecules. This relation for the chemically activated reaction rate constants is 14.7. The rate constants of the chemically activated reaction of SO_4_^•−^and ^•^OH are also correlated. There are some connections also between the rate constants of the low reactivity radicals, Cl_2_^•−^, CO_3_^•−^, and ClO^•^.

All radicals show higher reactivity with those molecules and parts within molecules (inner selectivity) that have higher activating electron density. This statement is also true for H^•^ reactions. The radicals may show selectively high reactivities with some organic molecules, e.g., Cl_2_^•−^ reacts with high rate constant with S-containing molecules.

The reactivity of Cl^•^, ^•^OH, SO_4_^•−^, Cl_2_^•−^, CO_3_^•−^, and ClO^•^ decreases with the decreasing reduction potential.

Correlations between rate constants of different radicals may help to establish the reaction mechanism, e.g., the relation between ^•^OH and H^•^ rate constants suggests a RAF/HAT mechanism for both radicals. Correlation between ^•^OH and SO_4_^•−^ chemically activated rate constants suggests that the RAF mechanism also occurs in SO_4_^•−^ reactions. Chemically activated rate constants can provide a correct basis for estimating the values of unknown rate constants.

## Figures and Tables

**Table 1:** One-electron reduction potentials *vs*. normal hydrogen electrode (NHE) of radical redox couples [13].

Radical	Reduction potentials *vs*. NHE, V
^•^OH/OH^−^	2.6
SO_4_^•−^/SO_4_^2−^	2.43
CO_3_^•−^, H^+^/HCO_3_^−^, pH 7	1.67
Cl_2_^•−^/2Cl^−^	2.1
Cl^•^/Cl^−^	2.6
ClO^•^/ClO^−^	1.39
H_aq_^+^/H^•^	−2.9

**Table 2:** Diffusion-controlled rate constants of radicals in reactions with organic molecules [5,15,16,17,18,19,20,21].

Radical	*k*_diff_, mol^−1^ dm^3^ s^−1^
^•^OH	1.1 × 10^10^
SO_4_^•−^	8.0 × 10^9^
CO_3_^•−^	7.3 × 10^9^
Cl_2_^•−^	7.3 × 10^9^
Cl^•^	~2 × 10^10^
ClO^•^	~1 × 10^10^
H^•^	2.9 × 10^10^

~: the number is approximately, not exactly.

**Table 3:** Rate constants of simple non-aromatic and aromatic molecules with reactive radicals, mol^−1^ dm^3^ s^−1^. The abbreviated names of the compounds are given in parentheses next to their names. In each box, the first value shows the measured or calculated rate constant, and the value below is the logarithm of the chemically activated rate constant.

Compound	^•^OH	SO_4_^•−^	CO_3_^•−^	Cl_2_^•−^	Cl^•^	ClO^•^	H^•^
Methanol (Me)	9.0 × 10^8 a/^ 8.95	7.0 × 10^6 a/^ 6.84	5.0 × 10^3 a/^ 3.70	2.0 × 10^4 b/^ 4.30	1.0 × 10^9 c/^ 9.00		2.7 × 10^6 a/^ 6.43
Ethanol (Et)	2.2 × 10^9 a/^ 9.43	3.5 × 10^7 a/^ 7.57	2.2 × 10^4 a/^ 4.34	9.0 × 10^4 b/^ 4.95	2.0 × 10^9 c/^ 9.35		1.7 × 10^7 a/^ 7.23
Isopropanol (Iso)	2.0 × 10^9 a/^ 9.39	6.0 × 10^7 a/^ 7.78	4.0 × 10^4 a/^ 4.60	1.6 × 10^5 b/^ 5.20	2.4 × 10^9 c/^ 9.43		7.0 × 10^7 a/^ 7.85
*tert*-BuOH (t-Bu)	6.2 × 10^8 a/^ 8.82	7.4 × 10^5 a/^ 5.87	<1.6 × 10^2 a/^ <2.20	2.6 × 10^4 b/^ 4.42	1.0 × 10^9 c/^ 9.01	negl. ^d/^	1.0 × 10^6 e/^ 6.00
Acetone (Ao)	1.1 × 10^8 a/^ 8.04		1.6 × 10^3 a/^ 3.20	1.6 × 10^3 b/^ 3.20	7.8 × 10^8 c/^ 8.90		2.0 × 10^6 f/^ 6.30
1,4-Dioxane (1,4-Di)	2.8 × 10^9 a/^ 9.58	4.3 × 10^7 a/^ 7.64		3.3 × 10^6 b/^ 6.52	4.4 × 10^9 c/^ 9.75	negl. ^d/^	1.0 × 10^7 a/^ 7.00
Tetrahydrofuran (Tetr)	4.0 × 10^9 a/^ 9.80	2.0 × 10^8 g/^ 8.31	4.9 × 10^4 d/^ 4.69	3.0 × 10^6 b/^ 6.48	2.6 × 10^9 f/^ 9.48		5.2 × 10^7 a/^ 7.71
Benzene (Be)	7.8 × 10^9 h/^ 10.42	1.8 × 10^9 i/^ 9.37	3.2 × 10^5 j/^ 5.50	<10^5 b/^ <5.00	6.0 × 10^9 c/^ 1.2 × 10^10^	3.2 × 10^4 d/^ 4.50	9.0 × 10^8 k/^ 8.97
Naphthalene (Naph)	9.3 × 10^9 l/^ 10.78	4.5 × 10^9 m/^ 10.01	7.4 × 10^6 l/^ 6.87	4.6 × 10^8 l/^ 8.69			3.4 × 10^9 a/^ 9.58
Toluene (T)	8.1 × 10^9 h/^ 10.49	2.4 × 10^9 i/^ 9.53	6.8 × 10^4 j/^ 4.83	<10^6 i/^ <6.00	1.8 × 10^10 c/^ 11.26	1.6 × 10^4 d/^ 4.20	1.8 × 10^9 k/^ 9.30
Fluorobenzene (Bef)	5.7 × 10^9 h/^ 10.07	9.8 × 10^8 i/^ 9.05				4.8 × 10^4 d/^ 4.68	1.5 × 10^9 k/^ 9.19
Chlorobenzene (ClB)	5.6 × 10^9 h/^ 10.06	1.5 × 10^9 i/^ 9.27	2.7 × 10 ^5 j/^ 5.43	<10^6 b/^ <6.00	1.8 × 10^10 c/^ 11.26	4.8 × 10^4 d/^ 4.68^/^	6.4 × 10^8 k/^ 8.81
Nitrobenzene (NB)	3.5 × 10^9 h/^ 9.71	≤10^6 i/^ <6.00	1.4 × 10^4 j/^ 4.14	negl. ^b/^	5.6 × 10^9 c/^ 9.89	2.6 × 10^3 d/^ 3.42	9.4 × 10^8 k/^ 8.98
Benzoic acid, neutral (Ban)	1.9 × 10^9 h/^ 9.361			≤10^6 e/^ <6.00	1.8 × 10^10 b/^ 11.26	5.2 × 10^3 d/^ 3.72	9.2 × 10^8 k/^ 8.964
Benzoic acid, anion (Baa)	5.9 × 10^9 h/^ 10.10	1.2 × 10^9 i/^ 9.15		2 × 10^6 e/^ 6.30	1.4 × 10^10 c/^ 10.62	<3 × 10^6 d/^ <6.47	1.3 × 10^9 a/^ 9.13
Phenol (Ph)	8.4 × 10^9 h/^ 10.55	6.2 × 10^9 i/^ 10.44	3.0 × 10^8 j/^ 8.49	2.8 × 10^8 b/^ 8.36	1.1 × 10^10 c/^ 10.301	8.0 × 10^6 d/^ 6.30	1.7 × 10^9 k/^ 9.26
Aniline, cation (Anc)	5.1 × 10^9 h/^ 9.98			1.2 × 10^7 e/^ 7.08		3.3 × 10^5 d/^ 5.52	4.0 × 10^8 a/^ 8.60
Aniline, neutral (Ann)	8.6 × 10^9 h/^ 10.60	7.7 × 10^9 i/^ 11.31	5.0 × 10^8 a/^ 8.73	6.8 × 10^8 b/^ 8.88	2.7 × 10^10 c/^	1.1 × 10^10 d/^ 10.39	1.9 × 10^9 k/^ 9.31
1,3,5-Trimethoxybenzene (TMB)	8.1 × 10^9 a/^ 10.49	2.0 × 10^9 i/^ 9.43		2.7 × 10^9 b/^ 9.63	1.1 × 10^10 c/^ 10.39	1.4 × 10^9 d/^ 9.21	~3.0 × 10^9 a/^ 9.52
Fumaric acid, neutral (Fum)	6.0 × 10^9 a/^ 10.12			1.2 × 10^5 b/^ 5.08	~3.3 × 10^9 c/^ 9.60		8.0 × 10^8 a/^ 8.91
Anisole (Ani)	5.4 × 10^9 a/^ 10.00	4.9 × 10^9 i/^ 10.10	2.8 × 10^5 j/^ 5.45	1.6 × 10^8 b/^ 8.21		3.3 × 10^6 d/^ 6.54	2.0 × 10^9 a/^ 9.33
*p*-Cresol (p-Cres)	9.2 × 10^9 h/^ 10.75	5.8 × 10^9 i/^ 10.32	1.5 × 10^8 j/^ 8.19		1.8 × 10^10 c/^ 11.26	1.5 × 10^6 d/^ 6.18	1.8 × 10^9 a/^ 9.28
Benzaldehyde (Ba)	2.6 × 10^9 h/^ 9.53	7.1 × 10^8 i/^ 8.89				3.2 × 10^5 d/^ 5.50	1.4 × 10^9 a/^ 9.14
Catechol, neutral (Catn)	1.1 × 10^10 a/^			5.7 × 10^8 b/^ 8.79	2.8 × 10^10 c/^	1.0 × 10^7 d/^ 7.00	
Resorcinol, neutral (Rn)	1.2 × 10^10 a/^				1.4 × 10^10 c/^ 10.67	1.0 × 10^7 d/^ 7.00	
Hydroquinone, neutral (Hq)	1.0 × 10^10 h/^ 11.04		2.3 × 10^9 j/^ 9.53	1.2 × 10^9 b/^ 9.16		1.0 × 10^7 d/^ 7.00	1.3 × 10^9 a/^ 9.14
4-Chlorophenol, neutral (4-ClPh)	7.6 × 10^9 a/^ 10.39		1.9 × 10^8 j/^ 8.29			1.3 × 10^6 d/^ 6.11	
p-Aminophenol, cation (p-Am)				4.0 × 10^9 b/^ 9.95		8.0 × 10^5 d/^ 5.90	
Dimethyl phthalate (Dmp)	3.7 × 10^9 n,o/^ 9.74	4.9 × 10^8 o/^ 9.27	<1 × 10^6 o/^ <6.00	1.4 × 10^7 o/^ 7.15	1.8 × 10^10 o/^ 11.26		
Diethyl phthalate (Dep)	3.4 × 10^9 o/^ 9.83	5.4 × 10^8 o/^ 8.71	<1 × 10^6 o/^ <6.00	1.1 × 10^7 o/^ 7.04	2.0 × 10^10 o/^		
Dibutyl phthalate (Dbp)	6.3 × 10 ^o,p/^ 10.17	5.5 × 10^8 o/^ 8.77	1.0 × 10^6 o/^ 6.00	1.1 × 10^7 p/^ 7.04	2.0 × 10^10 o/^		
Cysteine (Cys)	5.4 × 10^9 r/^ 10.01		2.1 × 10^8 a/^ 8.33	8.5 × 10^8 a/^ 8.98			
Bisphenol (Bis)	6.9 × 10^9 s/^ 10.26	4.5 × 10^9 t/^ 9.88	2.5 × 10^8 j/^ 8.41	5.8 × 10^8 b/^ 8.80	1.6 × 10^10 i/^ 10.90	2.2 × 10^8 d/^ 8.35	

^a/^ [7]; ^b/^ [19]; ^c/^ [23] ^d/^ [5]; ^e/^ [24]; ^f/^ [8]; ^g/^ [25]; ^h/^ [15]; ^i/^ [17]; ^j/^ [4]; ^k/^ [10]; ^l/^ [26]; ^m/^ [27]; ^n/^ [18]; ^o/^ [28,29]; ^p/^ [30]; ^r/^ [31]; ^s/^ [32]; ^t/^ [33]; ~: the number is approximately, not exactly.

**Table 4:** Rate constants of pharmaceuticals and miscellaneous molecules with reactive radicals, mol^−1^ dm^3^ s^−1^. The abbreviated names of the compounds are given in parentheses next to their names. In each box, the first value shows the measured or calculated rate constant, the value below is the logarithm of the chemically activated rate constant.

Compound	^•^OH	SO_4_^•−^	CO_3_^•−^	Cl_2_^•−^	Cl^•^	ClO^•^	H^•^
Ibuprofen (Ibu)	7.4 × 10^9 a/^ 10.36	3.8 × 10^9 b/^ 9.85	1.2 × 10^6 c/^ 6.08	<5 × 10^6 d/^ <6.70	2.0 × 10^10 e/^	5.5 × 10^6 f/^ 6.74	4.0 × 10^9 a/^ 9.66
Ketoprofen (Ket)	4.6 × 10^9 g/^ 9.90		3.9 × 10^8 c/^ 8.60				
Diclofenac (Dic)	8.1 × 10^9 g/^ 10.49	6.7 × 10^9 b/^ 10.61	7.8 × 10^7 c/^ 7.89	1.2 × 10^9 d/^ 9.16	3.8 × 10^10 e/^	3.5 × 10^8 f/^ 8.56	
Acetaminofen (Ace)	7.1 × 10^9 g/^ 10.30	3.0 × 10^9 b/^ 9.68	1.7 × 10^8 c/^ 8.23	4.4 × 10^8 d/^ 8.67	1.3 × 10^10 e/^ 10.57	4.6 × 10^8 f/^ 8.69	
Trimethoprim (TMP)	7.4 × 10^9 g/^ 10.36	6.5 × 10^9 b/^ 10.54	2.4 × 10^7 c/^ 7.38	1.9 × 10^9 d/^ 9.41	1.8 × 10^10 e/^ 11.26	2.8 × 10^6 f/^ 6.45	
Atenolol (Ate)	7.2 × 10^9 h/^ 10.32	5.1 × 10^9 h/^ 10.15	6.3 × 10^6 h/^ 6.80	4.1 × 10^8 h/^ 8.64	1.7 × 10^10 h/^ 11.05	8.7 × 10^7 h/^ 7.94	
Propranolol (Pro)	1.1 × 10^10 h/^	4.8 × 10^9 h/^ 10.08	2.0 × 10^8 h/^ 8.31	1.8 × 10^9 h/^ 9.38			
Metoprolol (Met)	8.0 × 10^9 h/^ 10.46	5.1 × 10^9 h/^ 10.15	3.7 × 10^6 h/^ 6.57	3.6 × 10^8 h/^ 8.57	1.7 × 10^10 h/^ 11.05	1.3 × 10^8 h/^ 8.12	
Gemfibrozil (Gem)	5.5 × 10^9 i/^ 10.04	7.1 × 10^8 b/^ 8.89	4.1 × 10^6 c/^ 6.61	2.9 × 10^8 d/^ 8.48	1.8 × 10^10 e/^ 11.26	7.7 × 10^8 f/^ 8.93	
Carbamazepine (Car)	6.0 × 10^9 j/^ 10.12	1.9 × 10^9 b/^ 9.40	3.3 × 10^6 c/^ 6.52	4.3 × 10^7 d/^ 7.63	3.3 × 10^10 e/^	7.6 × 10^7 f/^ 7.88	
Salicyclic acid (Sa)	1.0 × 10^10 l/^ 11.03	1.6 × 10^9 b/^ 9.30		2.1 × 10^8 d/^ 8.33			2.2 × 10^9 l/^ 9.38
Amoxicillin (Am)	6.7 × 10^9 m/^ 10.23	3.4 × 10^9 b/^ 9.77		1.6 × 10^9 n/^ 9.31	1.1 × 10^10 e/^ 10.39		
Ciprofloxacin (Cip)	6.1 × 10^9 m/^ 10.14	8.4 × 10^8 b/^ 8.97		2.2 × 10^8 d/^ 8.35	1.4 × 10^10 e/^ 10.67		
Ofloxacin (Of)	4.2 × 10^9 m/^ 9.83			3.5 × 10^8 d/^ 8.57	1.5 × 10^10 e/^ 10.78		
Tetracycline (Tet)	6.5 × 10^9 m/^ 10.20			1.2 × 10^9 d/^ 9.16	2.0 × 10^10 e/^		8.9 × 10^8 k/^ 8.96
Sulfamethoxazole (SMX)	6.4 × 10^9 m/^ 10.19	3.0 × 10^9 b/^ 9.68	2.8 × 10^8 c/^ 8.46	4.7 × 10^8 d/^ 8.70	3.5 × 10^10 e/^	<2 × 10^9 f/^ <9.44	
Naproxen (Nap)	8.6 × 10^9 o/^ 10.60	5.6 × 10^9 b/^ 10.27	5.6 × 10^7 c/^ 7.75	6.6 × 10^8 d/^ 8.86	2 × 10^10 e/^	<5.7 × 10^9 f/^ <10.41	
Metronidazole (Metr)	4.0 × 10^9 m/^ 9.80	2.7 × 10^9 b/^ 9.61	3.4 × 10^7 c/^ 7.53	1.2 × 10^8 d/^ 8.08	4.4 × 10^9 e/^ 9.75	<1 × 10^6 f/^ <6.00	
Erythromicin (Ery)	3.9 × 10^9 m/^ 9.78		8 × 10^7 c/^ 7.90		7.0 × 10^9 e/^ 10.03	4.6 × 10^9 f/^ 10.09	
Bezafibrate (Bez)	7.4 × 10^9 j/^ 10.35	1.9 × 10^9 j/^ 9.39	5.3 × 10^6 c/^ 6.72		1.0 × 10^10 e/^ 10.30	3.6 × 10^7 f/^ 7.56	
Clofibric acid (Clo)	5.5 × 10^9 r/^ 10.04	1.7 × 10^9 b/^ 9.33	1.0 × 10^7 c/^ 7.00	1.4 × 10^8 d/^ 8.15	5.5 × 10^9 e/^ 9.88		
2,4-Dichloro- phenoxyacetic acid, (2,4-D)	5.5 × 10^9 g/^ 10.04						1.4 × 10^9 k/^ 9.17
Triclosan (Tric)	5.6 × 10^9 m/^ 10.06	9.6 × 10^8 b/^ 9.03	4.2 × 10^7 c/^ 7.62	2.5 × 10^8 d/^ 8.41	2.8 × 10^10 e/^		
Atrazine (At)	2.4 × 10^9 p/^ 9.487	2.6 × 10^9 b/^ 9.58	4.0 × 10^6 d/^ 6.60	5.0 × 10^4 r/^ 4.70	6.9 × 10^9 e/^ 10.02	<10^6 f/^ <6.00	
Caffeine (Caf)	4.0 × 10^9 o/^ 9.80			9.3 × 10^8 d/^ 9.03	1.5 × 10^10 e/^ 10.78	1.4 × 10^9 f/^ 9.24	4.0 × 10^8 k/^ 8.61

^a/^ [34]; ^b/^ [17]; ^c/^ [4]; ^d/^ [19]; ^e/^ [23]; ^f^/ [5]; ^g/^ [18]; ^h/^ [35]; ^i/^ [36]; ^j^/ [37]; ^k/^ [7]; ^l/^ [38]; ^m/^ [39]; ^n/^ [40]; ^o/^ [41]; ^p/^ [25]; ^r/^ [42].

**Table 5:** Comparison of the reactivities of radicals.

Compound	^•^OH	SO_4_^•−^	CO_3_^•−^	Cl_2_^•−^	Cl^•^	ClO^•^	H^•^
*k*, small alcohols	6 × 10^8^–2 × 10^9^	10^6^–10^8^	10^3^–10^4^	10^4^–10^5^	10^9^–2 × 10^9^	negl.	10^6^–10^7^
*k*, simple aromatics	2 × 10^9^–8 × 10^9^	10^8^–8 × 10^9^	10^4^–4 × 10^8^	≤10^6^–2 × 10^9^	10^10^–2 × 10^10^	<10^4^–10^9^	4 × 10^8^–2 × 10^9^
Selectivity	aromatics		sulfites, amines	sulfites		amines, sulfites, phenols, methoxyben-zenes	aromatics
Mechanism	RAF/HAT	RAF/HAT	RAF/HAT/SET	SET/HAT	SET/RAF	RAF	RAF/HAT

## Data Availability

Not applicable.

## Abbreviations

The following abbreviations are used in this manuscript:

AOP	Advanced Oxidation Processes
NHE	Normal hydrogen electrode
HAT	H-atom transfer
SET	Single electron transfer
RAF	Radical adduct formation
